# Evaluation of renal near-infrared spectroscopy for predicting extubation outcomes in the pediatric intensive care setting

**DOI:** 10.3389/fped.2023.1326550

**Published:** 2024-01-19

**Authors:** Mustafa Colak, Gokhan Ceylan, Sevgi Topal, Ozlem Sarac Sandal, Gulhan Atakul, Ekin Soydan, Ferhat Sarı, Pinar Hepduman, Utku Karaarslan, Hasan Ağın

**Affiliations:** ^1^ Department of Paediatric Intensive Care Unit, Basaksehir Cam and Sakura City Hospital, Istanbul, Turkey; ^2^ Department of Paediatric Intensive Care Unit, Dr Behcet Uz Children's Disease and Surgery Training and Research Hospital, Health Sciences University, Izmir, Turkey; ^3^ Department of Medical Research, Hamilton Medical AG, Bonaduz, Switzerland

**Keywords:** extubation readiness test, mechanical ventilation, near-infrared spectroscopy, noninvasive monitoring, pediatric intensive care

## Abstract

**Background:**

In pediatric intensive care units, extubation failure following invasive mechanical ventilation poses significant health risks. Determining readiness for extubation in children can minimize associated morbidity and mortality. This study investigates the potential role of renal near-infrared spectroscopy (RrSO2) in predicting extubation failure in pediatric patients.

**Methods:**

A total of 84 patients aged between 1 month and 18 years, mechanically ventilated for at least 24 h, were included in this prospective study. RrSO2 levels were measured using near-infrared spectroscopy before and during an extubation readiness test (ERT). The primary outcome measure was extubation failure, defined as a need for reintubation within 48 h.

**Results:**

Of the 84 patients, 71 (84.6%) were successfully extubated, while 13 (15.4%) failed extubation. RrSO2 was found to be lower in the failed extubation group, also decrease in RrSO2 values during ERT was significantly greater in patients with extubation failure. ROC analysis indicated a decrease in ΔRrSO2 of more than 6.15% from baseline as a significant predictor of extubation failure, with a sensitivity of 0.984 and a specificity of 0.889.

**Conclusion:**

Monitoring changes in RrSO2 values may serve as a helpful tool to predict extubation failure in pediatric patients. Further multi-center research is warranted to improve the generalizability and reliability of these findings.

## 1 Introduction

Invasive mechanical ventilation is a life-saving practice commonly employed in pediatric intensive care units (PICUs) (1). However, it poses significant risks such as ventilator-associated pneumonia (VAP), ventilator-associated lung injury (VILI), patient self-induced lung injury (P-SILI), ventilator-associated diaphragm damage, and long-term exposure to narcotics and sedatives (2–4). Thus, it is crucial not to keep patients intubated longer than necessary. In the process of weaning patients from mechanical ventilation support, there are some accepted criteria that determine whether a patient's condition is suitable. These criteria include the patient being hemodynamically stable, capable of achieving adequate oxygenation, and performing gas exchange within acceptable limits (5). Determining the readiness of pediatric patients for extubation is critical to minimize morbidity and mortality associated with prolonged mechanical ventilation and extubation failure (6). Unfortunately, a significant proportion of patients who were once considered candidates for extubation require re-intubation after the extubation procedure. Rates of extubation failure in pediatric intensive care units have been reported to vary widely, from 2.7% to 22%, across studies that differ in patient populations and are influenced by the variability in study design and objectives. Despite the implementation of extubation criteria to evaluate the readiness for discontinuation of mechanical ventilation, there are instances where children may not be able to maintain effective spontaneous breathing after a planned extubation (7).

To prevent unsuccessful extubation and its associated complications, extubation readiness protocols are employed in pediatric care. However, despite the existence of various published protocols, there is a lack of standardized guidelines for integrating these practices into extubation protocols for the pediatric population. This absence of standardization can lead to significant variations in clinical practice and potential delays in weaning from invasive ventilation (8).

Near-infrared spectroscopy (NIRS) is a non-invasive technique used to continuously measure cerebral and regional oxygen saturation (rSO2). Initially used in the operating room to assess the decrease in cerebral blood flow during cardiopulmonary bypass, its use has since been extended to the critical care unit for patients at risk of multiple organ dysfunction or death (9). A correlation has been identified between the monitoring of renal-regional oxygen saturation (RrSO2) and invasive renal oxygen measurements during cardiac surgery (10). Moreover, an association has been observed between decreased rSO2 values during the extubation readiness test (ERT) and extubation failure in post-cardiac surgery patients (11). Additionally, studies involving neonates monitored after cardiac surgery have identified a significant decrease in renal NIRS values associated with extubation failure (12).

Despite the application of extubation readiness tests, extubation failure is still observed in pediatric patients. This means we need further tests or methods to avoid extubation failure. Specifically, in neonates and children monitored post-cardiac surgery, a reduction in renal NIRS levels during the preparation phase for extubation has been correlated with extubation failure. However, the relationship between renal NIRS values and extubation failure during ERT in critically ill pediatric patients other than cardiac surgery patients is not clear. This study aimed to examine the relationship between changes in RrSO2 and extubation outcomes. By doing so, we sought to shed light on the potential correlation and explore ways to minimize unnecessary intubations by reducing the risk of extubation failure.

## 2 Materials and method

### 2.1 Ethics

The study was conducted in compliance with the Helsinki Declaration, and the research protocol was approved by the ethics committee of Dr. Behcet Uz Children's Research and Training Hospital (no: 13399118-799). Informed consent was obtained from the patients' next of kin before enrollment.

### 2.2 Participants

The study included patients aged between 1 month and 18 years who had been mechanically ventilated with an endotracheal tube for at least 24 h before undergoing an extubation readiness test (ERT). Eligible patients were required to pass both the ERT and the cuff-leak test and exhibit no signs of upper airway obstruction. Exclusion criteria included patients who did not provide informed consent, those undergoing follow-up after cardiac surgery, patients with neuromuscular diseases, respiratory tract anomalies, renal agenesis or vascular malformations, and those with hemoglobinopathy or cyanide intoxication.

### 2.3 Procedure

The patients for whom extubation was planned were determined for the 9.00 pm round. Dexamethasone at a dose of 0.15 mg/kg was administered at both 00:00 and 06:00 am. The first visit was re-evaluated the next day and a cuff leak test was applied. A decision was made to extubate patients who met the criteria for extubation and successfully passed the cuff leak test. Enteral feeding was stopped at least 1 h before the extubation readiness test (ERT). At the commencement of the procedure, patients' sedatives were titrated until their State Behavioral Scale (SBS) scores were at either 0 or 1 (13). Subsequently, using an ultrasound machine, the patient's right kidneys were visualized with a 5–12 MHz frequency probe (HD 15, Philips Healthcare, Bothell, WA, USA) placed alongside the T12-L2 vertebra.

The probe of a near-infrared spectroscopy device (INVOSTM 5100, Somanetics Corporation, Troy, Michigan, USA) was then positioned on the ultrasound-visualized right kidney area. The HAMILTON MEDICAL C3 or C6 mechanical ventilator (Hamilton Medical, Bonaduz, Switzerland) was deployed either for manual adjustments of the expiratory trigger sensitivity (ETS), pressure ramp, and flow trigger or for automatic synchronization through embedded software.

Patient ID was entered in the NIRS device and recording started the NIRS device commenced recording RrSO2 values at 30-s intervals. After one hour of observation, ERT was started. During this phase, a spontaneous mode (PS) was employed with a positive end-expiratory pressure (PEEP) setting of 5 centimeters of water (cm H2O). The pressure support was adapted in relation to the internal diameter of the endotracheal tube (ETT), set at 10 cm H2O for ETT with a 3–3.5 mm internal diameter, 8 cm H2O for ETT with a 4–4.5 mm internal diameter, and 6 cm H2O for ETT with an internal diameter larger than 5 mm.

The extubation readiness test (ERT) was continued for 2 h. Throughout the extubation readiness test (ERT), patients were mandated to sustain a SpO2 above 95% and an expiratory tidal volume (VTe) of at least 5 ml/kg based on the predicted body weight. If the recorded respiration rate exceeded the acceptable target rate for the patient's age, or minimum SpO2 or VT is not sustained, the test was deemed unsuccessful. Age-related respiratory rates were classified as follows: 20–60 /min for 1 month to 6 months, 15–45 /min for 6 months to 2 years, 15–40 /min for 2–5 years, and 10–35 /min for patients older than 5 years (6, 14).

Baseline RrSO2 before ERT and RrSO2 values during ERT data were taken from the NIRS device. Data were analyzed with Invos Analytics Tool software (Version 1.2). Whole NIRS data containing one-hour before and during ERT were documented and average values for both phases were recorded.

Ninety-one extubation tests were performed on 84 patients. Extubation readiness test failed in seven patients. These patients were re-considered for extubation the next day and extubated after a successful ERT.

### 2.4 Statistical analysis

Statistical analyses were conducted using SPSS version 20 (IBM Corp., Armonk, NY) and JASP (JASP Statistics, Version 0.16.3, University of Amsterdam). The normality of the study data was assessed using the Kolmogorov–Smirnoff analysis. Participants' ages, length of stay on mechanical ventilator support, PIM 3 scores, respiratory and hemodynamic measures during ERT, and Renal-rSO2 values were stratified based on extubation success, and values were presented as median and 25–75 quantiles, and the Mann–Whitney *U*-test was employed to discern statistically significant differences between the groups with successful and unsuccessful extubation outcomes. The *p*-value of less than 0.05 was considered statistically significant.

We also calculated post-power of our study and found that sample sizes of 73 and 11 achieve 92% power to detect a difference of 1.10 between the group means with standard deviations of 1.0 and 1.00 at a significance level (alpha) of 0.05 using a two-sided *z*-test. These results assume that 5 sequential tests are made using the O’Brien-Fleming spending function to determine the test boundaries.

## 3 Results

**Figure 1:**
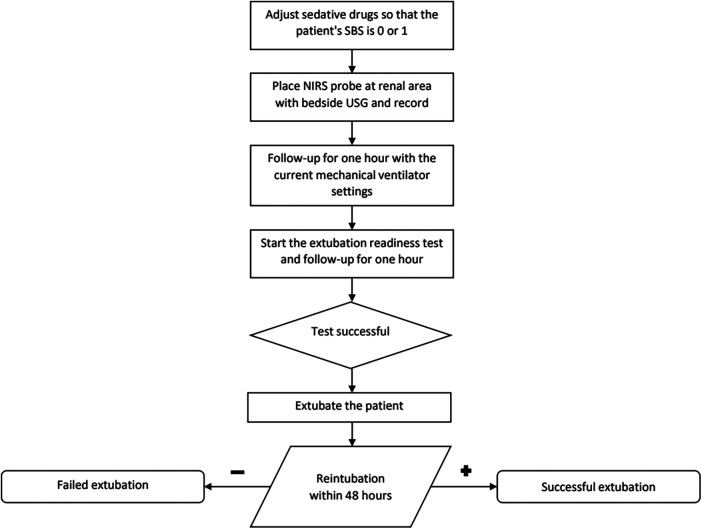
The study flow chart.

**Figure 2:**
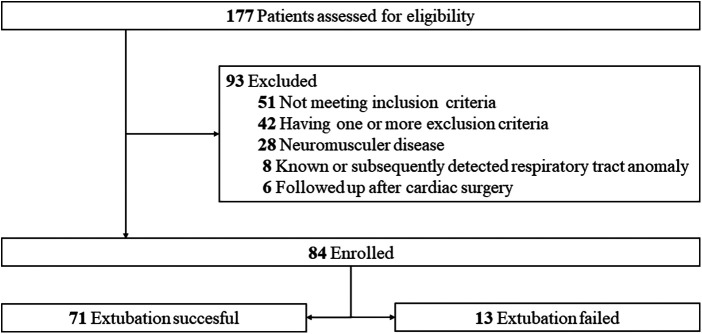
Study participants and excluded patients.

**Figure 3:**
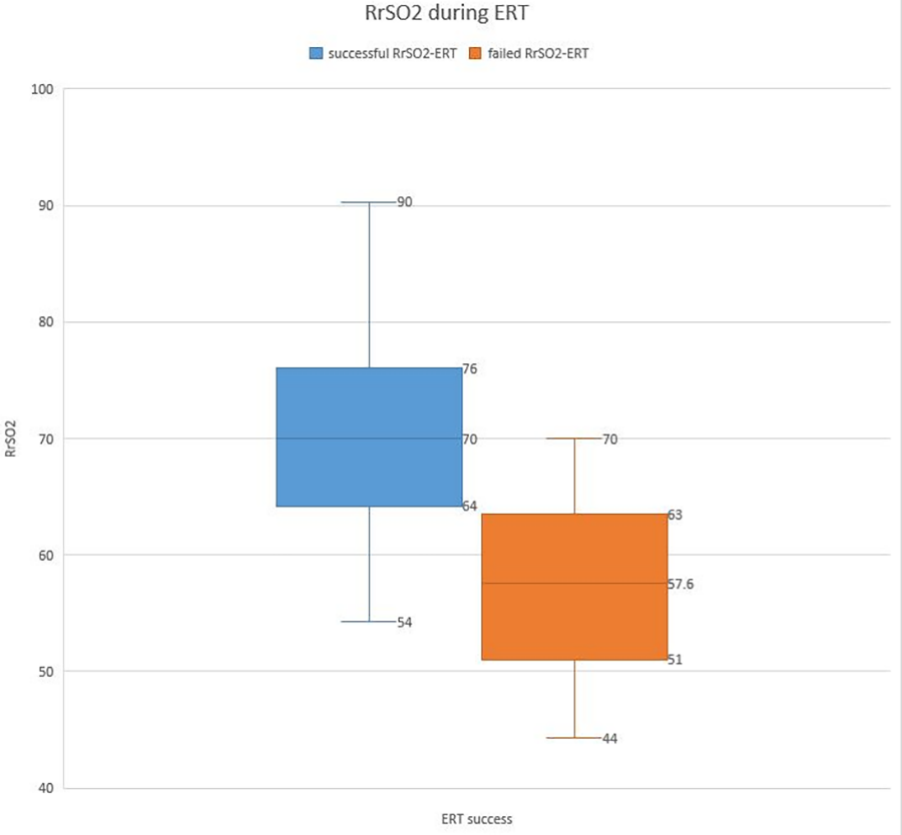
The RrSO2 during ERT.

**Figure 4:**
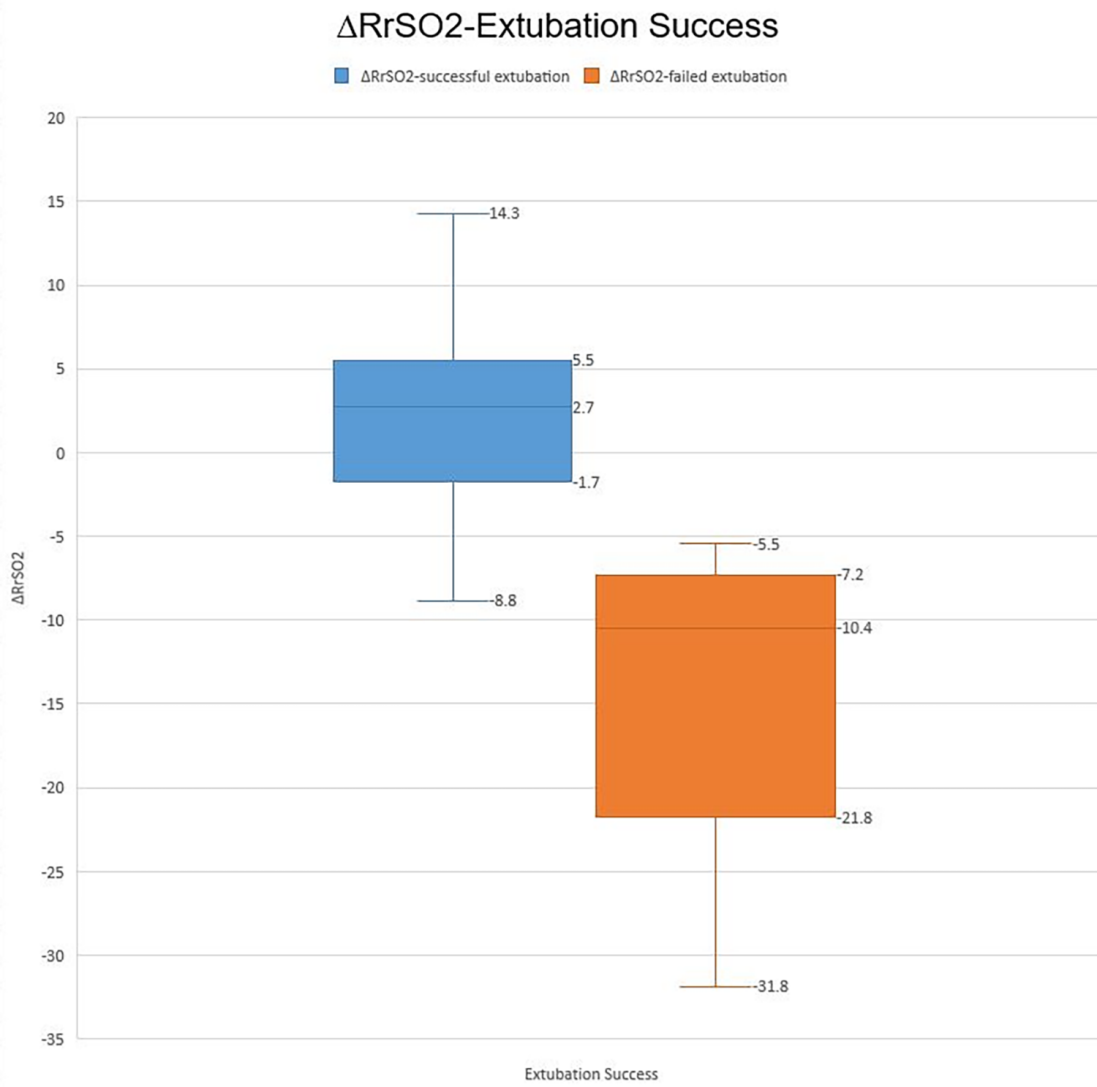
∆RrSO2-Extubation success.

A total of 84 patients were enrolled in the study, including 48 males and 36 females. The characteristics and reasons for intensive care unit hospitalization are shown in Table 1. Among the patients deemed suitable for extubation, a total of 91 ERT were conducted on 84 patients who successfully passed the cuff leak test. Of these, 77 patients were successfully extubated following the initial ERT. The remaining 7 patients underwent a second ERT the following day and were extubated following a successful outcome of this test. Of the participants, 71 (84.6%) were successfully extubated, while 13 (15.4%) failed extubation (Figures 1, 2). Participants were categorized according to extubation success, and significant differences were observed in age (months) and length of stay on mechanical ventilation, respiratory rates at the start of ERT, maximum respiratory rate during ERT, heart rate at the start of ERT, maximum heart rate during ERT and, heart rate at end of the ERT. During ERT, RrSO2 was found to be lower in the failed extubation group. No significant difference was observed between the groups in terms of the lowest oxygen saturation (SO_2_) values during the ERT, as well as in the partial pressure of oxygen (PO_2_) and carbon dioxide (PCO_2_) in the arterial blood gas taken at the end of the ERT. Not only the comparison RrSO2 values during the ERT but also comparison of the ΔRrSO2 values between patients who required reintubation and those who were successfully extubated revealed a significant decrease in ΔRrSO2 during ERT in the extubation failure group (−10.4 [(−21.8)–(−7.2)] vs. 2.7 [(−1.7)–(5.5)], as presented in Table 2 and Figures 3, 4. The NIRS values of the seven patients who did not pass the ERT on the first day were found to be: Before ERT Renal-rSO2 at 66 (58–71), during ERT RrSO2 at 54 (49–61), and ΔRrSO2 at 14 [(−19)–(−12.9)].

**Table 1:** Demographics of the patients at the PICU admission.

Age (months)	Median (IQR) 38.5 (6–42)
Gender	
Female	36
Male	48
PIM 3 score	1.55 (1.2–2.5)
Etiology	Number (%)
Pneumonia	51 (65.7)
Sepsis/septic shock	13 (12.8)
Status epilepticus	11 (11.4)
Metabolic disease	8 (8.5)
Drug poisoning	1 (1.4)

PIM 3, pediatric index of mortality 3.

**Table 2:** Characteristics and comparison of patients according to extubation success.

	Extubation successful (*n* = 71)	Extubation failed (*n* = 13)	*p* Value
Age (month)	26 (8–77)	5 (3.5–13)	0.002
Ventilation days	8 (6–9)	13 (10–16)	<0.001
gPIM 3 score	1.6 (1.2–2.5)	1.5 (1.2–3.4)	0.774
During ERT SO_2_ (%)			
Start of ERT	97 (96–99)	97 (96–98)	0.685
Max during ERT	98 (97–99)	97 (96–98)	0.140
Min during ERT	96 (95–97)	96 (95–98)	0.897
End of ERT	98 (95–98)	96 (95–98)	0.264
End of ERT PO_2_ (mmHg)	92 (85–98)	89 (83–96)	0.237
End of ERT PCO_2_ (mmHg)	42 (35–46)	44 (38–48)	0.465
During ERT pH	7.39 (7.38–7.41)	7.39 (7.37–7.41)	0.880
Respiratory rate (per min)			
Start of ERT	32 (24–37)	38 (35–41)	0.009
Max during ERT	36 (28–40)	42 (39–45)	0.002
Min during ERT	30 (22–36)	35 (30–38)	0.244
End of ERT	35 (27–41)	40 (37–42)	0.190
Heart Rate (per min)			
Start of ERT	108 (98–122)	124 (115–135)	0.002
Max during ERT	116 (105–128)	131 (122–142)	0.003
Min during ERT	107 (95–121)	115 (109–123)	0.289
End of ERT	110 (101–122)	128 (117–136)	0.002
Renal-rSO_2_			
Before ERT	69 (64–73)	65 (64–69)	0.353
During ERT	70 (64–76)	57.6 (51–63)	<0.001
ΔRrSO_2_	2.7 (−1.7), (5.5)	−10.4(−21.8), (−7.2)	<0.001

ERT, extubation readiness test; Max, maximum; Min, minimum; PCO_2_, partial carbon dioxide pressure in arterial blood gas; PO_2_, partial oxygen pressure in arterial blood gas; PIM 3, pediatric index of mortality 3; rSO_2_, regional oxygen saturation; SO_2_, oxygen saturation (pulse oximeter) ΔRrSO_2_, percentage change between before ERT Renal-rSO_2_ and During ERT Renal-rSO_2_ Values are represented as median and IQR 1 and 3 (25th–75th percentile) consecutively.

The ROC analysis conducted for ΔRrSO2 demonstrated that a decrease of more than 6.15% from baseline had a high predictive value for extubation failure, with a sensitivity of 0.944 and specificity of 0.769 (AOC: 0.965).

## 4 Discussion

Our observational study aimed to assess whether renal NIRS levels could function as an indicative factor for predicting the probability of extubation failure in pediatric patients. Successful implementation of this approach might result in decreased requirements for reintubation, consequently reducing the rates of mortality and morbidity linked to unsuccessful extubation in this specific patient group. The study compared the ΔRrSO2 values of patients who were successfully extubated to those who were reintubated. The extubation failure group had significantly lower ΔRrSO2 values during ERT (−10.4 [(−21.7)–(−7.2)] vs. 2.7 [(−1.7)–(5.5)], (*p* < 0.01). The study population underwent ROC analysis, and the cut-off value of −6.15% was determined for RrSO2. This value had a sensitivity of 0.944 and a specificity of 0.769.

Cerebral blood circulation is sensitive to changes in hydrogen ion levels, and acidosis due to increased CO2 levels in patients on respiratory support can cause cerebral artery vasodilation, leading to increased cerebral blood flow (CBF) and decreased blood flow to other organs (15, 16). A study using an animal model of hemorrhagic shock monitored cerebral and renal blood flow using NIRS and found that the reduction in renal blood flow occurred before the reduction in cerebral blood flow (17).

Changes in CBF play an important role in stabilizing breathing patterns in the central respiratory chemoreflex. The increase in CBF promotes diffusion of CO2 from cerebrospinal and cerebral extracellular fluids to the cerebral vasculature, resulting in a decrease in hydrogen ion concentration and suppressing pH decrease at the central chemoreceptors (18, 19). We think that, the patient did not experience tachypnea during ERT due to increased blood flow to the brain. However, the increase in cerebral blood flow resulted in decreased renal blood flow, causing a decrease in RrSO2 values. Foster et al. conducted a study on post-operative cardiac surgery patients and found that somatic rSO2 values decreased, but cerebral rSO2 values did not significantly change, which was similar to the results of this study (11).

Another on athletes also found a breakpoint in NIRS values of respiratory muscles during exercise, indicating a decrease in values when the respiratory compensation threshold was exceeded. Similar to this study, we demonstrated a decrease in renal NIRS values before the occurrence of the breakpoint (20).

In our study, the rate of extubation failure was found to be 15.4%, which is consistent with previous literature (7, 21). We observed a statistically significant difference in the median age between patients who passed the ERT and were successfully extubated vs. those who failed and were reintubated. The median age for reintubated patients was 5 months, while for successfully extubated patients it was 42 months. This highlights the importance of considering age as a potential factor when assessing the likelihood of reintubation after passing the ERT. Dexamatasone was administered to all patients who met the inclusion criteria and for whom extubation was decided. Numerous controversial studies have been conducted on the use of dexamethasone before or during the extubation of pediatric patients, specifically focusing on the reintubation rate. Nevertheless, the majority of these studies suggest a reduced occurrence of events related to upper airway obstruction in the dexamethasone group. A recent meta-analysis, incorporating 10 randomized controlled studies, concluded that corticosteroid use is deemed acceptable. As a result, we administered dexamethasone with the goal of minimizing these events, aiming to mitigate any potential impact on NIRS values within the study population (22, 23). Although our study aimed to investigate the relationship between RrSO2 levels and extubation failure in pediatric patients, we acknowledge several limitations that should be taken into account. First and foremost, our research was conducted at a single center, which may limit the generalizability of our findings to other populations and settings. Moreover, we acknowledge that there may be other confounding factors that contribute to the risk of extubation failure that we did not account for in our study design. Therefore, caution should be exercised in interpreting our results as a causal relationship between RrSO2 levels and extubation failure cannot be established without further experimental evidence. In order to improve the validity and applicability of the research findings, we suggest that future studies be conducted across multiple centers and increase the number of participants. It is essential to include patients who have undergone long-term mechanical ventilation, suffer from associated lung diseases, neuromuscular weakness after intensive care or related pathology, sedation or delirium issues, as well as congenital comorbidities such as tracheobronchomalacia, vocal cord dysfunction or diaphragmatic paralysis, as they significantly contribute to extubation failure. Additionally, there is still a contentious debate in the existing literature regarding the type of post-extubation treatments (such as oxygen therapy, high-flow nasal cannula, or nasal continuous positive airway pressure) that help patients achieve a successful extubation. The findings and limitations of this study should be considered in larger studies to advance this important observational study.

## 5 Conclusion

The use of RrSO2 monitoring during extubation in our study showed a significant decrease in RrSO2 values in patients who were unsuccessfully extubated, indicating its potential as a tool for identifying those at risk of extubation failure. Alongside ERT, we recommend monitoring RrSO2 before and during extubation and considering changes in ΔRrSO2 when making extubation decisions. Although our study had limitations, such as a small sample size and being conducted at a single center, future research, including multicenter studies, can provide more comprehensive understanding and improve the success rate of extubation in pediatric patients.

## Data Availability

The raw data supporting the conclusions of this article will be made available by the authors, without undue reservation.
