# Supplementary figures and images for: Evaluation of renal near-infrared spectroscopy for predicting extubation outcomes in the pediatric intensive care setting
Source: Front Pediatr. 2024 Jan 19;11:1326550. doi: 10.3389/fped.2023.1326550 (PMC10834679; doi:10.3389/fped.2023.1326550)

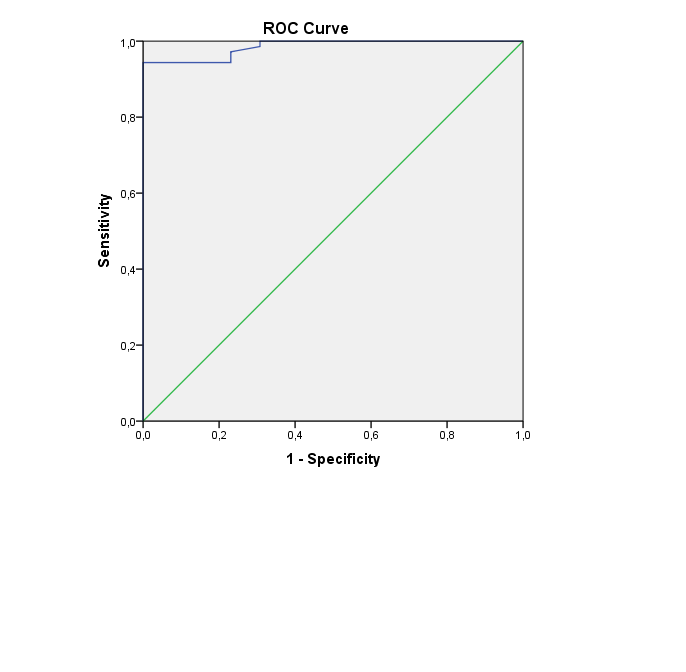

Supplement: Supplementary file 1 [file Image1.tiff]
